# Lost in translation? Information quality in pediatric pre-hospital medical emergencies with a language barrier in Germany

**DOI:** 10.1186/s12887-023-04121-y

**Published:** 2023-06-21

**Authors:** Frank Müller, Dominik Schröder, Jennifer Schäning, Sybille Schmid, Eva Maria Noack

**Affiliations:** 1 Department of General Practice, University Medical Center Göttingen, Humboldtallee 38, 37073 Göttingen, DE Germany; 2 Fire Department, City of Braunschweig, Brunswick, DE Germany

**Keywords:** Language barrier, Migrant, Refugee, Emergency medical service, Pediatric emergency, Documentation quality

## Abstract

**Background:**

In pediatric medical emergencies, paramedics and emergency physicians must often rely on the information of third parties, often caregivers, to gather information. Failing to obtain relevant information may lead to misinterpretation of symptoms and subsequent errors in decision making and clinical treatment. Thus, children and/or caregivers with limited proficiency of the locally spoken language may be at risk for medical errors. This study analyzes logs of rescue missions to determine whether paramedics could obtain essential information from German-speaking and foreign-language children and their caregivers.

**Methods:**

We conducted a secondary data analysis based on retrospective data on pediatric patients of four emergency medical services (EMS) stations in Northern Germany. We defined language discordance with communication difficulties as main exposure. We used documentation quality as outcome defined as existing information on (a) pre-existing conditions, (b) current medication, and (c) events prior to the medical emergency. Statistical analyses include descriptive statistics, simple regression and multivariable regression. As multivariable regression model, a logistic regression was applied with documentation quality as dependent variable and language discordance with communication difficulties as independent variable adjusted for age, sex and Glasgow Coma Scale (GCS).

**Results:**

Data from 1,430 pediatric rescue missions were analyzed with 3.1% (*n* = 45) having a language discordance with communication difficulties. Patients in the pediatric foreign-language group were younger compared to German-speaking patients. Thorough documentation was more frequent in German-speaking patients than in patients in the foreign-language group. Pre-existing conditions and events prior to the medical emergency were considerably more often documented in German-speaking than for foreign-language patients. Documentation of medication did not differ between these groups. The adjustment of sex, age and GCS in the multivariable analysis did not change the results.

**Conclusion:**

Language barriers are hindering paramedics to obtain relevant information in pediatric pre-hospital emergencies. This jeopardizes the safe provision of paramedic care to children who themselves or their caregivers are not fluent in German language. Further research should focus on feasible ways to overcome language barriers in pre-hospital emergencies.

**Trial registration:**

This is a retrospective secondary data analysis of a study that was registered at the German Clinical Trials Register (No. DRKS00016719), 08/02/2019.

**Supplementary Information:**

The online version contains supplementary material available at 10.1186/s12887-023-04121-y.

## Background

In recent decades, the linguistic heterogeneity in Western countries has considerably grown due to increasing migration and travel. In Germany, more than 2.3 million refugees sought protection between 2015 and 2020 [1, 2]. The war in Ukraine has resulted in the admission of an additional 1,000,000 refugees [3]. Furthermore, Germany is a destination for seasonal workers, especially from Eastern Europe, as well as for tourists and business travelers from all over the world [4]. Providing care to patients who are not proficient in the locally spoken language and are not familiar with navigating the local healthcare system is a great challenge. It has been shown in various healthcare settings that it negatively impacts medical outcomes if patients and healthcare providers do not speak a common language [5–7]. Likewise, studies have highlighted the importance of (professional) language interpretation to provide an equitable and high standard of care [8–12]. It is especially challenging to provide safe and high-quality health care to foreign-language children whose parents have also limited language proficiency of the common local language [13–16]. Language barriers in pre-hospital emergency care are especially challenging for various reasons: First, the care environment in emergency medical services (EMS) is unpredictable with a variety of patients and symptoms, mainly associated with traumatology or acute conditions in various locations introducing uncertainty in rescue missions [17]. Second, for many emergencies, it is key to understand patients’ current complaints and pre-existing conditions. Misunderstandings can lead to inaccurate initial assessment and an increased risk of incorrect clinical decisions and treatments. Language barriers have shown to delay transport to hospital [18, 19], to increase the likelihood of hospitalization [20] and (unnecessary) intubation and intensive care during rescue missions with trauma patients [21]. At the same time, possibility to interpret on site is usually very limited during rescue missions.

Children make up only a small percentage of pre-hospital emergency patients, but pediatric emergencies are special challenges for the treating paramedics and emergency physicians: anatomical and physiological peculiarities at different ages on the one hand, and children’s different emotional, cognitive, and communicative characteristics and skills on the other hand [22]. To care properly for a child, it is important to know the events that preceded the emergency call and possibly underlying health conditions, and to understand the symptoms.

In pediatric emergencies, paramedics and emergency physicians must often rely on the information of third parties, often caregivers, to gather information. However, if the child, their caregivers, and the paramedics do not share a common language, medical history taking may be impeded. Even basic information about physical conditions, such as age, height, weight, which might be important for medication dosages, may be impossible to obtain. Additionally, a trusting atmosphere should be created for the child and, if present, for an accompanying person. Addressing the child in an adequate manner, the paramedics should explain to them everything that is happening and involve their caregivers in the treatment [23]. Language barriers in pediatric emergency care may thus cause misinterpretation of symptoms and lacking to obtain and to provide information on the emergency situation. This may result in inadequate treatment and in a negative experience of children and caregivers [24, 25]. While hospitals or medical practices may have interpreting resources such as multilingual staff [26], at emergency scenes qualified language interpreters are generally unavailable to bridge language barriers. A language barrier was found to be the most frequent obstacle when providing emergency care to refugee children [27]. Despite the challenging communication in pediatric emergencies and the consequences language barriers can have, there is insufficient research on this topic in Germany.

The present study aims to determine whether paramedics could collect and document essential information from foreign-language children and their caregivers by analyzing logs of pediatric medical emergencies with and without a language barrier.

## Methods

This is a cross-sectional study based on a retrospective secondary data analysis of depersonalized rescue mission logs of four EMS stations in Germany. These rescue mission logs (called “DIVI-Protokoll” as they are standardized by the Deutsche Interdisziplinäre Vereinigung für Intensiv- und Notfallmedizin e.V., the German Interdisciplinary Association for Intensive and Emergency Medicine) are used for the legally correct documentation of prehospital rescue missions [28, 29]. The data collection was part of the DICTUM-Rescue project [30], where a fixed-phrase translator app was developed to enhance communication with foreign-language patients [31, 32]. We excluded emergency cases where this app was used from the analyses as in a pilot study the use of the app was associated with higher perceived overall quality of communication [33].

### Setting

The study area lies in the Federal State of Lower Saxony, Germany. It comprises the rural county of Helmstedt (approximately 91,000 inhabitants) and partly the municipality of Braunschweig (approximately 250,000 inhabitants). There are four regional hospitals, two of which have pediatric clinics. In addition, there is a large psychiatric hospital in the region. The EMS stations in Königslutter, Wendhausen and Braunschweig are served by the Malteser Hilfsdienst gGmbH (Order of Malta Volunteers), the station in Helmstedt is belongs to the county. Each station has two to five ambulances and deploys 2,500 to 4,000 rescue missions per year. The EMS station in Braunschweig is one out of five EMS stations that serve certain catchment areas of the city and are provided and staffed by different organizations/institutions. Within the catchment area of the study EMS station in Braunschweig there is a reception facility for refugees. Patients with limited German language proficiency treated by EMS in this region include migrants and refugees residing in the region, transit travelers (especially along the motorway), tourists and seasonal workers in industry and agriculture. EMS is generally covered by the patients’ statutory or private health insurances, as having health insurance is mandatory for residents in Germany. For many citizens of the European Union, and some other European countries (Serbia, Switzerland, Norway) expenses for EMS services are covered within the framework of the European Health Insurance Card (EHIC). People without legal residency status and/or without health insurance status are entitled to emergency treatment [34]. German EMS follows the concept of “bringing the hospital to the patients “ with advanced emergency care providing by trained paramedics and/or emergency physicians on scene [35].

### Data collection

For every EMS rescue mission, a standardized rescue mission log is used to document initial assessment, treatment, and transport. We extracted data on all rescue missions from January 15^th^, 2019 to March 10^th^, 2021 at the three EMS stations in the county of Helmstedt (786 days) and from May 15^th^, 2019 to December 31^st^, 2020 (597 days) for the Braunschweig EMS station. The three EMS stations of the county of Helmstedt used a digital log (software CEUS® Rettungsdienst, CKS Systeme GmbH, Meppen, Germany), so data was exported digitally. The EMS station in Braunschweig used paper–pencil-based logs. Here, a trained study nurse and experienced paramedic performed manual data extraction.

### Main exposures and covariates

We extracted the following patient-related information from all logs: patients’ age and sex, current medical condition (including Glasgow Coma Scale (GCS) [36], National Advisory Committee for Aeronautics-Score (NACA-Score) [37], and need for ventilation), preliminary diagnosis and paramedics’ description of the emergency as free text. Moreover, we extracted information regarding the rescue operation itself: dispatch of emergency physician (yes/no), time-en-route (time to reach patient, i.e. time spent driving between departure from rescue station and arrival at emergency scene, in minutes), on-scene time (time spent on emergency scene, i.e. time between arrival at and departure from emergency scene, in minutes), and time to destination (time between departure from emergency and hospital, in minutes).

We defined pediatric patients as ≤ 18 years old and the main exposure “foreign language speaker” as either or both (a) a documented language barrier encountered during the rescue mission or documentation that patients and / or caregivers spoke another language than German, which made communication difficult or impossible or (b) communication was supported by a third person who was interpreting. On the standardized logs there is no specific field to indicate rescue missions with language barriers; paramedics document challenges in communicating with patients, such as a language discordance, in their description of the emergency (as free text). We analyzed these text fields manually for all pediatric rescue missions and also extracted the spoken language, if available.

To assess the impact of patient-provider language discordance on the quality of obtained information, FM and JS reviewed all pediatric cases with regard to documented information on (a) pre-existing conditions, (b) current medication, and (c) prior events that may have led to the medical emergency. We developed these criteria by aggregating the SAMPLE history scheme, a set of information that should be obtained in pre-hospital emergency care. These are: symptoms, allergies, medication, past medical history, **l**ast oral intake, **e**vents prior to incident [38]. For our study, we considered the description of patients’ symptoms to be not as relevant, as these can typically be conveyed non-verbally (e.g. injuries) or can be at least roughly recognized by paramedics by observing certain signs (e.g. pain, dyspnea). We collapsed the items “allergies” and “past medical history” to “pre-existing conditions” and adopted “medication” and “events prior to incident” as is. Lack of obtaining and communicating these information to hospital care providers have been shown to impact outcomes among trauma patients [39]. For this in-depth review of open text fields, we established criteria in advance, as to when we considered information to have been obtained. These were, for example, that “prior events that may have led to the medical emergency” refers to a time span of 24 h prior to the incident. For example, if a free text would indicate, that a person had used dimenhydrinate some hours before the emergency, this would qualify that information on “prior events” have been obtained but not “current medication” as the latter stands for ongoing drug treatments.

If both paramedics and emergency physicians were dispatched to a patient, typically two logs were completed. In these cases, one log is often kept brief and is referring to the other, regardless that both teams have access to similar information. Accordingly, if for an emergency more than one log was available, we combined information from all protocols.

Cases considered uncertain were discussed among the reviewers. As an additional quality control, a random sample of 5% of the cases was double-checked by a researcher (EMN) who was not involved in the original coding. We found a rater-interrater reliability of 97.3%.

### Statistics

We characterize our samples using descriptive statistics to assess the characteristics of patients served in rescue missions stratified by language proficiency with absolute and relative frequencies, mean and standard deviation (SD). Percentages of cases where relevant information has been documented are illustrated in bar graphs stratified for German-speaking and foreign-language patients with corresponding 95% confidence intervals (CI).

We perform multivariable analysis using binary logistic regression models with a logit link function to assess the association of language proficiency (German-speaking patients [ref] vs. foreign-language patients) on documentation of (a) pre-existing conditions, (b) current medication, and (c) prior events that may have led to the medical emergency while adjusting for age, sex and GCS. We report adjusted odd’s ratios (aOR) with aOR > 1 indicating worse documentation in foreign-language patients compared to German-speaking patients. A comprehensive table with aOR for all covariables is shown in a supplement file (Supplement file 1). We used SPSS 28 (IBM, Armonk, NY, USA) for all analyses and plotted the bar graph using Graph Pad Prism 9.5.0. (GraphPad Software, San Diego, CA).

### Research ethics

This study received approval from the Research Ethics Board of the University Medical Center Göttingen (9/9/18). The original study is registered at the German Clinical Trials Register (No. DRKS00016719). Cooperation and data usage agreements with all participating EMS providers, the Municipality of Braunschweig and the District of Helmstedt were signed.

## Results

### Sample characteristics

**Fig. 1:**
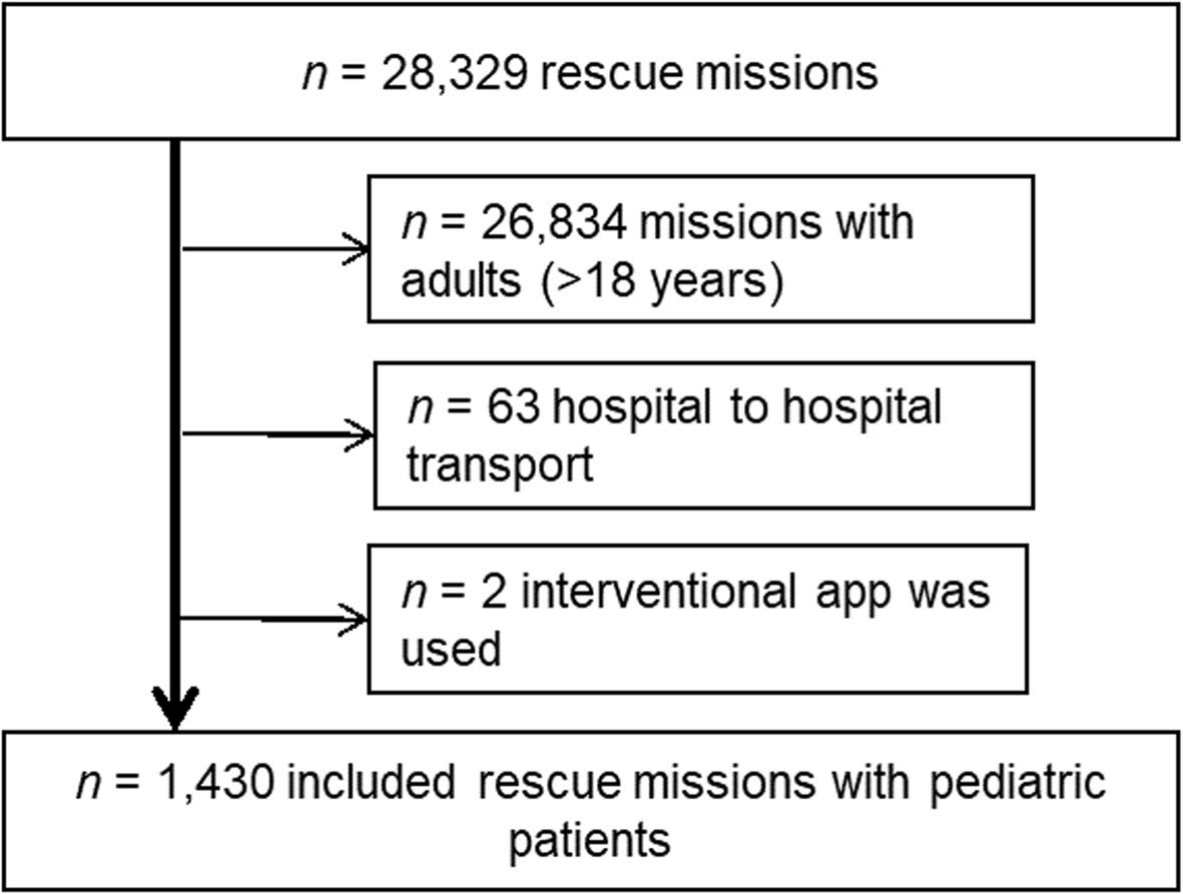
Flowchart of included rescue missions

During the study period, we recorded 1,430 rescue missions with pediatric patients (5.1% of all rescue missions) that we subsequently analyzed. Figure 1 shows the flowchart of included rescue missions. On average, 52.2 (SD 19.0) pediatric patients were treated per month.

In 3.1% (*n* = 45) of pediatric emergency cases, a language discordance with the patient and/or their guardians was noted. In 25 (55.5%) of these cases, the primary spoken language was documented. The most prevalent languages were Arabic (*n* = 6), Bosnian/Croatian/Serbian (*n* = 4), and Turkish (*n* = 3).

Table 1 shows information on patients’ demographics and characteristics of rescue missions. Pediatric foreign-language patients were on average 3 years younger than German-speaking pediatric patients, with a considerably lower proportion of teenagers aged 13–18 in the foreign-language group (21.3% vs. 47.7%). The sex ratio was balanced in both groups (female 45.5% vs. 49.0%). The timespan to reach the patient (time-en-route) and the timespan paramedics spent on the emergency scene (on-scene time) did not differ between foreign-language and German-speaking patients (7.4 min vs. 8.1 min and 18.2 min vs. 19.3 min). Dispatch at nighttime was more often among foreign-language patients (45.5% vs. 31.8%). The transport to hospital took foreign-language patient on average four minutes longer than German-speaking patients.

**Table 1:** Patients’ demographics and characteristics of rescue missions

Rescue missions		Unit^a^	All *N* = 1,430	German-speaking *n* = 1,385	Foreign-language *n* = 45
Patients’ demographics					
Sex	male	n (%)	716 (51.1)	692 (51.0)	24 (54.5)
	female	n (%)	685 (48.9)	665 (49.0)	20 (45.5)
	other	n (%)	1 (0.1)	1 (0.1)	0 (0)
Age	years	Mean (SD)	10.2 (6.2)	10.3 (6.2)	7.3 (5.5)
	< 1	n (%)	40 (2.8)	38 (2.7)	2 (4.4)
	1- < 6	n (%)	410 (28.7)	393 (28.4)	17 (37.8)
	6- < 13	n (%)	310 (21.7)	294 (21.2)	16 (35.6)
	13–18	n (%)	670 (46.9)	660 (47.7)	10 (22.2)
Characteristics of rescue missions					
EMS Station	Braunschweig	n (%)	439 (30.7)	434 (31.3)	5 (11.1)
	Wendhausen	n (%)	347 (24.3)	331 (23.9)	16 (35.6)
	Königslutter	n (%)	238 (16.6)	226 (16.3)	12 (26.7)
	Helmstedt	n (%)	406 (28.4)	394 (28.4)	12 (26.7)
Time-en-route (min)^b^		Mean (SD)	8.1 (4.8)	8.1 (4.8)	7.4 (4.2)
On-scene time (min)^c^		Mean (SD)	19.2 (20.3)	19.3 (20.6)	18.2 (9.7)
Time to destination (min)^d^		Mean (SD)	18.4 (10.2)	18.2 (10.2)	22.4 (8.6)
Dispatch at nighttime (8PM-7AM)		n (%)	436 (32.3)	416 (31.8)	20 (45.5)
Fall / Wintertime (Oct-Mar)		n (%)	692 (49.0)	671 (49.1)	21 (46.7)
Emergency physician present		n (%)	303 (29.2)	294 (29.5)	9 (22.5)
Patient rejected transport / further care		n (%)	94 (6.6)	93 (6.7)	1 (2.2)

^a^Missing sex n = 28, missing dispatch at nighttime n = 78, missing time fall/wintertime n = 19; ^b^Time-en-route: The timespan between departure from rescue station and arrival at rescue scene; ^c^On-scene time: timespan paramedics spent on the emergency scene; ^d^Time to destination: timespan between departure from emergency scene to hospital

In our sample, slight injuries were most common (26.9%) followed by psychiatric disorders (13.8%) and respiratory disorders (11.7%). Foreign-language patients were more likely to have a cardiovascular disorder as preliminary diagnosis than German-speaking patients (Table 2).

**Table 2:** Medical characteristics of patients

		Unit	All *N* = 1,430	German-speaking *n* = 1,385	Foreign-language *n* = 45
Initial Assessment					
Glasgow Coma Scale (GCS)^a^		Mean (SD)	14.5 (2.0)	14.5 (2.0)	14.8 (1.3)
	15	n (%)	1,246 (88.9)	1,202 (88.6)	44 (97.8)
	10–14	n (%)	98 (7.0)	98 (7.2)	0 (0.0)
	< 10	n (%)	58 (4.1)	57 (4.2)	1 (2.2)
NACA-Score^a^		Mean (SD)	2.5 (1.0)	2.5 (1.0)	2.6 (0.8)
	1	n (%)	110 (15.6)	109 (16.1)	1 (3.4)
	2	n (%)	244 (34.6)	234 (34.6)	10 (34.5)
	3	n (%)	286 (40.6)	270 (39.9)	16 (55.2)
	4	n (%)	44 (6.2)	43 (6.4)	1 (3.4)
	5–7	n (%)	21 (3.0)	20 (3.0)	1 (3.4)
Need for ventilation		n (%)	10 (0.7)	9 (0.6)	1 (2.2)
Preliminary Diagnosis					
Neurological disorders		n (%)	161 (11.3)	157 (11.3)	4 (8.9)
Cardiovascular disorders		n (%)	70 (4.9)	63 (4.5)	7 (15.6)
Respiratory disorders		n (%)	167 (11.7)	159 (11.5)	8 (17.8)
Metabolic disorders		n (%)	21 (1.5)	19 (1.4)	2 (4.4)
Psychiatric disorders		n (%)	197 (13.8)	195 (14.1)	2 (4.4)
Abdominal disorders		n (%)	80 (5.6)	76 (5.5)	4 (8.9)
Gynecological and obstetric disorders		n (%)	7 (0.5)	6 (0.4)	1 (2.2)
Other disorders		n (%)	85 (5.9)	80 (5.8)	5 (11.1)
Injuries	None	n (%)	907 (63.4)	877 (63.3)	30 (66.7)
	Slight	n (%)	385 (26.9)	373 (26.9)	12 (26.7)
	Moderate	n (%)	114 (8.0)	112 (8.1)	2 (4.4)
	Severe	n (%)	24 (1.7)	23 (1.7)	1 (2.2)

^a^Missing GCS *n* = 28, missing NACA-Score *n* = 725

### Quality of documented information

**Fig. 2:**
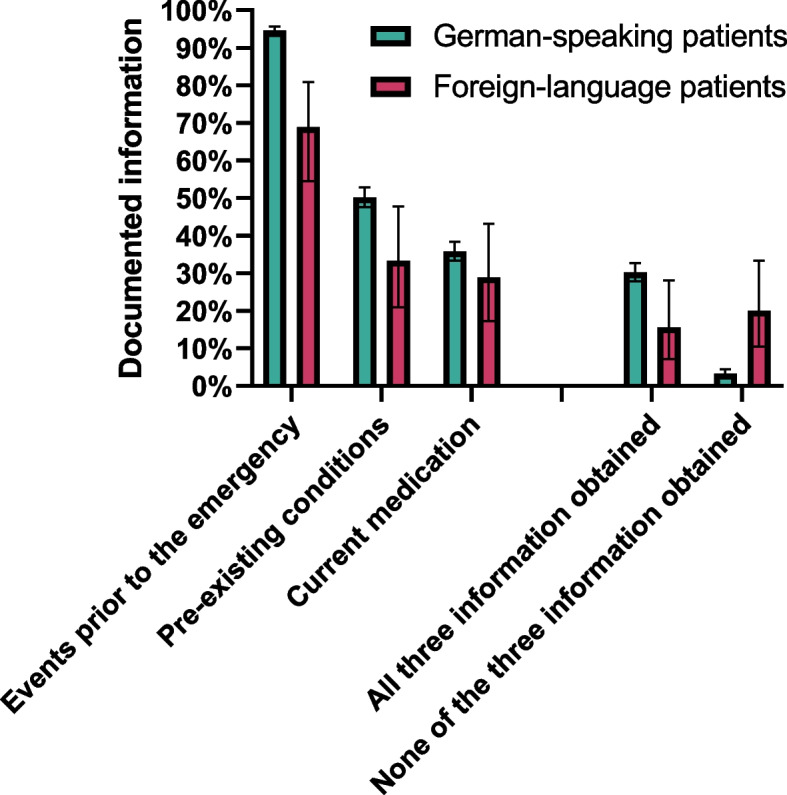
Documented information on pediatric patients: foreign-language and German-speaking patients. German-speaking patients: missing demographic values *n* = 20

Obtained and documented information differed considerably between foreign-language patients and German-speaking patients: While information on pre-existing conditions were found in half of all cases documented for German-speaking patients, this information was documented for only 31.1% of foreign-language patients (*p* = 0.015). Similarly, events that led to the medical emergency were only noted in 68.9% of the cases with foreign-language patients compared to 94.1% of German-speaking patients (*p* < 0.001). Current medication did not differ between both groups. None of the surveyed information was documented in 3.2% of cases for German-speaking patients but in 20.0% of foreign-language patients (Fig. 2).

After adjusting for age, sex, and GCS, foreign language-patients were still more likely to have an inadequate documentation on pre-existing conditions and events prior to the medical emergency (*p* < 0.001) (Table 3). The regression coefficients of all independent variables included in the regression models and odd ratios can be found in the Supplement Table S1.

**Table 3:** Documented information

	All *n* = 1,410	Foreign-language patients *n* = 45	German-speaking (ref) *n* = 1,365	Multivariable Model^a^
	n (%)	n (%)	n (%)	OR (95% CI)
Pre-existing conditions	697 (49.4)	14 (31.1)	683 (50.0)	2.01 (1.04 – 3.93)
Current medication	493 (35.0)	11 (24.4)	482 (35.3)	1.86 (0.91 – 3.80)
Events prior to the medical emergency	1,327 (94.1)	31 (68.9)	1,296 (94.9)	7.97 (4.01 – 15.82)

^a^Generalized linear model adjusted for age, sex, GCS. OR for foreign-language patients, German proficient as reference. An OR > 1 indicates less documentation. German-speaking patients: missing demographic values *n* = 20. OR on included covariables are shown in supplement file S1

## Discussion

In this study, we analyzed logs of 1,430 pediatric rescue missions in Germany. Our main findings are as follows: First, in 3.1% of cases a language barrier and / or a language other than German spoken by patients and guardians was documented, including cases where a third person was interpreting. Second, in these cases the documentation of pre-existing conditions and events leading to the emergency was considerably more often lacking compared to those without a documented language barrier. This finding remains valid when controlled for age, sex and GCS.

Off note, the proportion of foreign-language pediatric patients was considerably higher compared to emergency patients of all ages with limited German proficiency [40, 41]. This may be partly explained by the lower mean age of migrants in Germany [42]. Besides, previous research has shown that foreign-language population in Germany uses EMS for other medical conditions than German-speaking population [40, 41, 43].

To our knowledge, this is the first study examining the impact of language barriers on documentation quality among pediatric patients in an EMS setting.

In addition, our results show that documentation could also be improved for German-speaking patients, despite existing standardized procedures for emergencies requiring paramedics to obtain certain information in every emergency, i.e. primary survey following the ABCDE-approach (ABCDE: Airway, Breathing, Circulation, Disability, Exposure [44]) and medical history taking following the SAMPLE-scheme [38]). Previous research has highlighted an overall poor documentation quality in EMS in Europe: In a retrospective analysis of 1,935 emergency protocols of a German EMS district, the quality of documentation and healthcare was found to be deficient in multiple cases. In only less than a quarter of cases, all items of the SAMPLE scheme for medical history taking had been documented [45]. Similarly, internal audits of EMS in a region in Austria showed that less than half of the logs were sufficiently completed [46].

Although limited documentation quality is common, our results show that language barriers are particularly associated with poor documentation quality, suggesting that paramedic struggle to obtain relevant information in pediatric medical emergencies when a language barrier is present. While standard operating procedures (SOPs) may improve quality of documentation in EMS [47], these are of little help when medical history taking is impeded by a language barrier.

Lacking information on medical history, regardless if it is due to language barriers or poor documentation or both, may impact not only the clinical decision-making of the emergency responders, but also further treatment in hospital [39]: anything that is not documented is potentially not passed onto subsequent healthcare providers. The handover between paramedics and the emergency department staff often takes place under time pressure in a stressful environment which bears a risk of harm or adverse events for the patient [48]. Underuse of interpreters is common in pediatric emergency departments [49, 50] and has various reasons [51]. This eventually worsens the chance that an information deficit is caught up among pediatric patients with language barriers. Furthermore, multilingual pediatric providers that could gap language barriers are rare in Germany [52].

Some of the 3-year paramedic training curricula in Germany comprise courses in “Medical English”. In some cases, it may be helpful in using English as a third language to communicate with foreign-language patients. Given the linguistic diversity of patients, with most common languages spoken from Eastern Europe and the Middle East, English language skills may help in just a minority of cases. There is no data on what foreign languages paramedics speak beside those learned at school.

Technical solutions, such as video or telephone interpreting services may improve patient-paramedic communication but are difficult to use in time-critical medical emergencies. Continuously improving machine translation may be used in commonly used languages but are often inaccurate in less common languages [53]. The translator app developed in the project affiliated with this study [31] could provide a suitable approach to improve communication with foreign-language patients [33]. The app can be used to collect a medical history addressing children and their caregivers in appropriate wording and could thus also lead to complete documentation. Furthermore, the app allows that posed questions and responses from patients and/or their caregivers can be logged and obtained information can be transferred to the emergency protocol.

### Limitations and strengths

The main limitation of our work involves the use of quality analyses of the documented pediatric rescue mission logs as a proxy for communication quality. From this, we can neither infer about the quality of medical care nor know how successful and satisfactory patients and paramedics perceived the communication.

We cannot ascertain whether information was obtained from patients or their caregivers but not documented, e.g. due to time constraints or a paramedic just forgetting to make a note. However, if communication is perceived as difficult, paramedics may be inclined to document more precisely to support subsequent treatment. If obtained information cannot be verified or double-checked, they might also be inclined to note a language barrier to avoid any suspicion of malpractice and protect themselves from potential litigation. While documentation has a high priority in healthcare in Germany and is subject to quality monitoring and is required by law, documentation quality may vary [45, 46]. Still, documentation can be considered a rather good proxy for assessing which information has been obtained.

We reviewed 1,430 pediatric rescue missions; nonetheless, the number of 45 analyzed cases with language discordance is a considerable limitation. There might have been rescue missions with foreign-language patients and parents that were not identifiable. For example, in cases of paramedics that spoke the language of the child or their guardians, and thus a language barrier was not reported. These cases would therefore be included in the German-speaking group. Moreover, we analyzed a data set from only four EMS stations served by two institutions. Organizational frameworks and quality standards may differ slightly among providers and regions.

As to the digitized paper protocols, it is possible that misinformation due to, for example, poor handwriting or atypical abbreviations might have introduced a bias. However, the study nurse who digitalized the paper protocols is a trained paramedic with many years of professional experience in the participating EMS stations. This makes this bias rather unlikely.

A strength of the study is the large and complete sample with a long survey period covering all seasons. The proportion of pediatric emergencies in our sample is with 5.1% consistent with another German study [54] and comparable of those described in the literature suggesting a representative data set [55].

Our study period comprised both timespans before and during the SARS-CoV-2-pandemic. This is important as the number of rescue service missions decreased after the lockdown started mid-March 2020 and the patient population changed, e.g. patients were considerably older and there were less patients with respiratory diseases [56, 57]. Also, pediatric emergency healthcare utilization reportedly declined considerably [58]. In our study, we cannot be completely certain if COVID-19 pandemic and respective mitigation measures may have affected our findings.

## Conclusions

Language barriers are hindering paramedics to obtain relevant information in pediatric pre-hospital emergencies. This jeopardizes a safe provision of paramedic care for foreign-language children. Further research should focus on feasible ways to overcome language barriers in pre-hospital emergencies.

## Supplementary Information

**Additional file 1: Supplement Table S1.** Generalized linear model on documented information.

## Data Availability

The datasets used and analyzed during the study are not publicly available due to the decision of the research ethics board but can be obtained from the authors upon reasonable request within a data sharing agreement.

## Abbreviations

COVID-19: Coronavirus disease 2019
EHIC: European Health Insurance Card
EMS: Emergency medical services
GCS: Glasgow coma scale
NACA-Score: National advisory committee for aeronautics-score
OR: Odds ratio
SARS-CoV-2: Severe acute respiratory syndrome coronavirus 2
SD: Standard deviation
SOP: Standard operating procedure

## References

[1] UNHCR, UNHCR Population Statistics. 2020. http://popstats.unhcr.org/en/overview. Accessed 4 May 2020.

[2] Bundesamt für Migration und Flüchtlinge. Das Bundesamt in Zahlen 2019. 2020. https://www.bamf.de/SharedDocs/Anlagen/DE/Statistik/BundesamtinZahlen/bundesamt-in-zahlen-2.https://www.bamf.de/SharedDocs/Anlagen/DE/Statistik/BundesamtinZahlen/bundesamt-in-zahlen-2019-asyl.pdf Accessed 13 Apr 2020.

[3] Mediendienst Integration. Ukrainische Flüchtlinge: Zahlen und Fakten. 2022. https://mediendienst-integration.de/migration/flucht-asyl/ukrainische-fluechtlinge.html. Accessed 17 Apr 2023.

[4] Statistisches Bundesamt (Destatis). Landwirtschaftliche Betriebe, Landwirtschaftlich genutzte Fläche, Arbeitskräfte, Arbeitsleistung. 2019. https://www-genesis.destatis.de/genesis/downloads/00/tables/41141-0113_00.csv. Accessed 11 Nov 2022.

[5] Cohen AL, Rivara F, Marcuse EK, McPhillips H, Davis R (2005). Are language barriers associated with serious medical events in hospitalized pediatric patients?. Pediatrics.

[6] Bauer AM, Alegría M (2010). Impact of patient language proficiency and interpreter service use on the quality of psychiatric care: a systematic review. Psychiatr Serv.

[7] Holman H, Müller F, Bhangu N, Kottutt J, Alshaarawy O (2022). Impact of Limited English proficiency on the diagnosis and awareness of diabetes: the National Health and Nutrition Examination Survey, 2003–2018. Diabetes Care.

[8] Ashton LM (2012). Caring for patients in any language: does it matter?. Nursing.

[9] Hadziabdic E, Albin B, Heikkilä K, Hjelm K (2010). Healthcare staffs perceptions of using interpreters: a qualitative study. Prim Health Care.

[10] Flores G (2005). The impact of medical interpreter services on the quality of health care: a systematic review. Med Care Res Rev.

[11] Karliner LS, Jacobs EA, Chen AH, Mutha S (2007). Do professional interpreters improve clinical care for patients with limited English proficiency? A systematic review of the literature. Health Serv Res.

[12] Führer A, Brzoska P (2022). Die relevanz des dolmetschens im gesundheitssystem. [The importance of language interpretation in the health care system]. Gesundheitswesen.

[13] Ciupitu CC, Babitsch B (2011). Why is it not working? Identifying barriers to the therapy of paediatric obesity in an intercultural setting. J Child Health Care.

[14] Ullrich S, Briel D, Nesterko Y, Hiemisch A, Brähler E, Glaesmer H (2016). Verständigung mit Patienten und Eltern mit Migrationshintergrund in der stationären allgemeinpädiatrischen Versorgung. [Communication with migrant patients and their parents in inpatient general pediatric care]. Gesundheitswesen.

[15] Dai X, Ryan MA, Clements AC, Tunkel DE, Links AR, Boss EF, Walsh JM (2021). The effect of language barriers at discharge on pediatric adenotonsillectomy outcomes and healthcare contact. Ann Otol Rhinol Laryngol.

[16] Zamor RL, Vaughn LM, McCann E, Sanchez L, Page EM, Mahabee-Gittens EM (2022). Perceptions and experiences of latinx parents with language barriers in a pediatric emergency department: a qualitative study. BMC Health Serv Res.

[17] Sundström BW, Dahlberg K (2012). Being prepared for the unprepared: a phenomenology field study of swedish prehospital care. J Emerg Nurs.

[18] Grow RW, Sztajnkrycer MD, Moore BR (2008). Language barriers as a reported cause of prehospital care delay in Minnesota. Prehosp Emerg Care.

[19] Perera N, Birnie T, Ngo H, Ball S, Whiteside A, Bray J (2021). I’m sorry, my english not very good”: tracking differences between language-barrier and non-language-barrier emergency ambulance calls for out-of-hospital cardiac arrest. Resuscitation.

[20] Lee ED, Rosenberg CR, Sixsmith DM, Pang D, Abularrage J (1998). Does a physician-patient language difference increase the probability of hospital admission?. Acad Emerg Med.

[21] Bard MR, Goettler CE, Schenarts PJ, Collins BA, Toschlog EA, Sagraves SG, Rotondo MF (2004). Language barrier leads to the unnecessary intubation of trauma patients. Am Surg.

[22] Karutz H, DʼAmelio R, Pajonk F-G (2012). Psychologische aspekte pädiatrischer notfallsituationen. Notf Med Up2date.

[23] Rielage T, Biederbick F (2019). Besonderheiten bei der Notfallversorgung von pädiatrischen Patienten. Retten.

[24] Tate RC, Kelley MC (2013). Triage in the tower of babel: interpreter services for children in the prehospital setting. Pediatr Emerg Care.

[25] Steinberg EM, Valenzuela-Araujo D, Zickafoose JS, Kieffer E, DeCamp LR (2016). The “Battle” of managing language barriers in health care. Clin Pediatr (Phila).

[26] Lundin C, Hadziabdic E, Hjelm K (2018). Language interpretation conditions and boundaries in multilingual and multicultural emergency healthcare. BMC Int Health Hum Rights.

[27] Nijman RG, Krone J, Mintegi S, Bidlingmaier C, Maconochie IK, Lyttle MD, von Both U (2021). Emergency care provided to refugee children in Europe: RefuNET: a cross-sectional survey study. Emerg Med J.

[28] Messelken M, Schlechtriemen T, Arntz HR, Bohn A, Bradschetl G, Brammen D (2011). Der Minimale Notfalldatensatz MIND3. Notarzt.

[29] Deutsche Interdisziplinäre Vereinigung für Intensiv- und Notfallmedizin (DIVI). DIVI-Notfalleinsatzprotokoll 6.1. https://www.divi.de/images/Dokumente/220308-divi-notfall-einsatzprotokoll-6.1.pdf. Accessed 9 Jan 2023.

[30] Noack EM, Kleinert E, Müller F (2020). Overcoming language barriers in paramedic care: a study protocol of the interventional trial ‘DICTUM rescue’ evaluating an app designed to improve communication between paramedics and foreign-language patients. BMC Health Serv Res.

[31] Noack EM, Schulze J, Müller F (2021). Designing an app to overcome language barriers in the delivery of emergency medical services: participatory development process. JMIR Mhealth Uhealth.

[32] Müller F, Hummers E, Schulze J, Noack EM. Nutz- und Bedienbarkeit einer App zur Überwindung von Sprachbarrieren im Rettungsdienst. [Usability of an app to overcome language barriers in paramedic care]. Notf Rett Med. 2021:1–7. 10.1007/s10049-021-00913-w. 10.1007/s10049-021-00913-w PMC8251687 34230808

[33] Müller F, Schröder D, Noack EM (2023). Overcoming language barriers in paramedic care with an app designed to improve communication with foreign-language patients: nonrandomized controlled pilot study. JMIR Form Res.

[34] Act on benefits for asylum seeker (Asylbewerberleistungsgesetz), Bundesgesetzblatt, Part I. 1997;(57):2022–6. https://www.gesetze-im-internet.de/asylblg/BJNR107410993.html. Accessed 19 June 2023.

[35] Al-Shaqsi S (2010). Models of international Emergency Medical Service (EMS) systems. Oman Med J.

[36] Gabbe BJ, Cameron PA, Finch CF (2003). The status of the Glasgow coma scale. Emerg Med (Fremantle).

[37] Schlechtriemen T, Burghofer K, Lackner CK, Altemeyer KH (2005). Validierung des NACA-Score anhand objektivierbarer parameter. Notf Rett Med.

[38] Marx JA, Hockberger RS, Walls RM, Adams J, Rosen P (2010). Rosen’s emergency medicine: concepts and clinical practice.

[39] Carter AJE, Davis KA, Evans LV, Cone DC (2009). Information loss in emergency medical services handover of trauma patients. Prehosp Emerg Care.

[40] Müller F, Hummers E, Noack EM (2020). Medical characteristics of foreign language patients in paramedic care. Int J Environ Res Public Health.

[41] Müller F, Noack EM (2023). Einfluss von Sprachbarrieren auf die Notrufabfrage: Darstellung von Qualitätsindikatoren und Einsatzanlässen [Impact of language barriers on emergency rescue dispatch: quality indicators and emergency characteristics]. Notarzt.

[42] Statistisches Bundesamt (Destatis). Bevölkerung und Erwerbstätigkeit. Bevölkerung mit Migrationshintergrund – Ergebnisse des Mikrozensus 2020 (Endergebnisse). Fachserie 1 Reihe 2.2. 2022. https://www.destatis.de/DE/Themen/Gesellschaft-Umwelt/Bevoelkerung/Migration-Integration/Publikationen/Downloads-Migration/migrationshintergrund-endergebnisse-2010220207004.pdf. Accessed 5 Dec 2022.

[43] Keizer E, Senn O, Christensen MB, Huibers L (2021). Use of acute care services by adults with a migrant background: a secondary analysis of a EurOOHnet survey. BMC Fam Pract.

[44] Soar J, Nolan JP, Böttiger BW, Perkins GD, Lott C, Carli P (2015). European Resuscitation Council Guidelines for Resuscitation 2015: sect. 3. Adult advanced life support. Resuscitation.

[45] Klein M, Schröder H, Beckers SK, Borgs C, Rossaint R, Felzen M (2022). Dokumentations- und Behandlungsqualität im Rettungsdienst: eine retrospektive Analyse von Einsatzprotokollen in der Stadt Aachen. [Quality of documentation and treatment in the non-physician staffed ambulance: a retrospective analysis of emergency protocols from the city of Aachen]. Anaesthesiologie.

[46] Neumayr A, Golger P, Schwaiger D, Schinnerl A, Karl A, Baubin M (2023). Audits zur Dokumentationsqualität im Rettungsdienst – ein Muss!. Notf Rett Med.

[47] Francis RCE, Schmidbauer W, Spies CD, Sörensen M, Bubser F, Kerner T (2010). Standard operating procedures as a tool to improve medical documentation in preclinical emergency medicine. Emerg Med J.

[48] Dúason S, Gunnarsson B, Svavarsdóttir MH (2021). Patient handover between ambulance crew and healthcare professionals in icelandic emergency departments: a qualitative study. Scand J Trauma Resusc Emerg Med.

[49] Sina B, Noemi G, Myriam G, Ursula F, Anne J, Jabeen F (2022). The use of intercultural interpreter services at a pediatric emergency department in Switzerland. BMC Health Serv Res.

[50] Hartford EA, Anderson AP, Klein EJ, Caglar D, Carlin K, Lion KC (2019). The use and impact of professional interpretation in a Pediatric Emergency Department. Acad Pediatr.

[51] Granhagen Jungner J, Tiselius E, Pergert P (2021). Reasons for not using interpreters to secure patient-safe communication - A national cross-sectional study in paediatric oncology. Patient Educ Couns.

[52] Müller F, Holman H, Hummers E, Schröder D, Noack EM (2022). Multilingual competencies among ambulatory care providers in three german Federal States. BMC Prim Care.

[53] Patil S, Davies P (2014). Use of google translate in medical communication: evaluation of accuracy. BMJ.

[54] Leibinger S (2012). Pädiatrische Notfälle im Rettungsdienst. Retten.

[55] Eich C, Russo SG, Heuer JF, Timmermann A, Gentkow U, Quintel M, Roessler M (2009). Characteristics of out-of-hospital paediatric emergencies attended by ambulance- and helicopter-based emergency physicians. Resuscitation.

[56] Müller F, Hummers E, Jablonka A, Schmidt T, Noack EM (2022). Auswirkung des COVID-19-Lockdowns auf Rettungseinsätze. [Impact of the COVID-19 lockdown on emergency medical service operations]. Notf Rett Med.

[57] Naujoks F, Schweigkofler U, Lenz W, Blau J, Brune I, Lischke V, et al. Veränderungen der rettungsdienstlichen Einsatzzahlen in einer Metropolregion während der ersten COVID-19-pandemie-bedingten Kontaktbeschränkungsphase. [Changes in number of emergency medical service deployments in an urban area during the first COVID-19 pandemic-related contact restriction phase]. Notf Rett Med. 2021:1–9. 10.1007/s10049-021-00875-z. 10.1007/s10049-021-00875-z PMC8063581 33935590

[58] Happle C, Dopfer C, Wetzke M, Scharff AZ, Mueller F, Dressler F, et al. Covid-19 related reduction in paediatric emergency healthcare utilization – a concerning trend. 2020. 10.1186/s12887-020-02303-6 PMC7475725 32894080

