# Supplementary material for: Lost in translation? Information quality in pediatric pre-hospital medical emergencies with a language barrier in Germany
Source: BMC Pediatr. 2023 Jun 21;23:312. doi: 10.1186/s12887-023-04121-y (PMC10283249; doi:10.1186/s12887-023-04121-y)
Supplement: Supplementary file 1 — Additional file 1: Supplement Table S1. Generalized linear model on documented information. [file 12887_2023_4121_MOESM1_ESM.docx]

# Supplementary Material

Supplement Table S1: Generalized linear model on documented information.

|  | | Pre-existing conditions | | Current medication | | Immediate events that lead to the medical emergency | |
| --- | --- | --- | --- | --- | --- | --- | --- |
|  |  | OR (95% CI) | p | OR (95% CI) | p | OR (95% CI) | p |
| Language | Foreign-language | 2.01 (1.04 - 3.93) | 0.039 | 1.86 (0.91 - 3.80) | 0.091 | 7.97 (4.01 - 15.82) | <0.001 |
|  | German | Ref. |  | Ref. |  | Ref. |  |
| Sex | Female | 0.73 (0.59 - 0.95) | 0.005 | 0.90 (0.72 - 1.13) | 0.371 | 1.02 (0.65 - 1.62) | 0.923 |
|  | Male | Ref. |  | Ref. |  | Ref. |  |
| Age in years (continuous) | | 1.07 (1.05 - 1.09) | <0.001 | 1.00 (0.98 - 1.02) | 0.988 | 1.03 (0.99 - 1.06) | 0.180 |
| GCS (continuous) | | 0.94 (0.89 - 0.99) | 0.032 | 0.97 (0.91 - 1.02) | 0.228 | 0.97 (0.85 - 1.11) | 0.642 |

GCS=Glasgow Coma Scale. An odd’s ratio >1 indicates less documentation.
